# Supplementary material for: Predicting death by the loss of intestinal function
Source: PLoS One. 2020 Apr 14;15(4):e0230970. doi: 10.1371/journal.pone.0230970 (PMC7156097; doi:10.1371/journal.pone.0230970)
Supplement: S1 File — (DOCX) [file pone.0230970.s001.docx]

STANDARD ROSE AND MUELLER LAB BANANA FOOD RECIPE

The laboratory is a controlled environment with respect to temperature and so on, and we must take care that the food given the flies is equally uniform in quality. Most of the adult phenotype is determined by the larval stages, and the larvae feed, bathe, and live in the banana food. It is therefore absolutely critical that the food be consistent, particularly when the flies are to be assayed in an experiment. Limited variation in banana ripeness & quality, slight burning (&c) can be tolerated for routine stock maintenance, but should be avoided as much as possible for experimental-generation flies. The ideal banana is ripe but not rotting, with full yellow color and the first hints of browning spots.

INGREDIENTS

| Cook Size | **4.4 Liters** | **6.6 Liters** |
| --- | --- | --- |
| **STEP 1** |  | |
| Distilled water | 4.4 Liters | 6.6 Liters |
| Agar | 66.7 grams | 100 grams |
| **STEP 2** |  |  |
| Bananas unpeeled | 600 grams | 900 grams |
| Distilled water | 267 ml | 400 ml |
| Light Karo syrup | 1 & 1/3 scoop * | 2 scoops * |
| Dark Karo syrup | 1 & 1/3 scoop * | 2 scoops * |
| Barley Malt | 2 scoops * | 2 scoops * |
| **STEP 3** |  | |
| Distilled water | 307 ml | 460 mL |
| 95% Ethanol | 107 ml | 160 mL |
| Yeast | 160 grams | 240 grams |
| **Step 4** |  | |
| 95% Ethanol | 104 ml | 156 mL |
| p-hydrobenzoic acid | 10.4 grams | 15.6 grams |
| * A "scoop" refers to the ice-cream scooper used in the Rose Lab, which is 55 mL, volumetrically | | |

DIRECTIONS

STEP 1

Start by measuring the water & agar and combining them in the cook pot with a stir-bar (a large one) and put the hot-plate on "high". Don't turn the stirring speed too high or the stir-bar will come off-center. Allow the pot to boil; this may take better than an hour, so you should start the cook while still attending to other things. The Bunsen burner may be used at this stage to speed up the boiling, but even at this stage over-boiling and burning are possible. Do not leave the lab with the flame on.

STEP 2

When the agar-water has boiled vigorously for at least 5 minutes you may proceed with step two; do not start this step too early, or else the mixture will congeal into nasty lumps and the bananas will oxidize. For the small (4.4L) cook one blender is sufficient, but two are necessary for the 6.6L cook. Add the water to the blender(s), which should be well cleaned, and then the weighed bananas one by one while blending on low. Make sure the bananas are smoothly blended in by switching to a high setting. Then add the other ingredients (karo & barley malt). Mix everything well and then add to the boiling agar-water. Do not wash the blenders. Using a wooden spoon to scrub the areas of the pot outside of the stir-bar's reach (don't knock stir-bar off center) and rotate the whole pot slightly help prevent burned spots which slow down re-boiling.

STEP 3

While it was critical in step one to allow the mixture to boil, the third step may be started before the food re-boils. Add most of the water to one of the blenders (at least 3/4), saving a bit in the graduated cylinder, and rinse it out before pouring it into the other blender (it only takes one) to get most of the residual banana mix out. Add ethanol, and, gradually while blending, the yeast. Blend until all particles of yeast are dissolved (5-10 minutes); too short and the yeast will precipitate out, too long and it will start growing in the blender. Use the bit of water you saved to rinse out the blender and add it to the cook. Allow the final mixture to boil for at least five minutes. As the food boils at the final stage it will foam up and overflow if the lid is on; you must watch the food as it nears a boil. Generally, it is best to take the lid off when the food comes to a boil and let it boil for at least five minutes. It is absolutely critical that the food boil after the yeast has been added.

STEP 4 anti-fungal solution

Remove the food and allow it to cool -- a cold water bath speeds this up. Mix up the

ethanol and hydrobenzoic acid and cover it to prevent evaporation. The food should be

stirred occasionally while cooling. When the temperature hits 48oC the anti-bacterial solution should be added, and mixed in thoroughly before pouring.

STEP 5 - pouring

Vials: For most purposes, we require vials for egg-collection/larval rearing. The standard depth in an 8-dram vial is about 2cm (or 6mL), which is well in excess of the amount eaten by the larvae; within reason, any level of food above about 1cm is adequate. A 6.6L cook will make about 41/2 racks of each. food if carefully poured; a 4.4L cook will make about 3 racks. Vials which are only for temporary feeding of adults (transfer vials) can be poured low.

Plates: Plates are usually poured up to the upper lip of a 15x100mm petri dish. A cook will produce about 12 - 14 plates / liter. Plates are almost always needed in the lab, so if you have extra food pour plates.

STEP 6 - cooling & storage

Putting the food in front of the fan will speed its cooling and help prevent stray flies from getting into it. Egg-collection vials should be covered with the screening and plates should not be left out for flies to lay on. When vials are room temperature (1/2 - 1 hour), they should be inverted with paper towel on top into a new & clearly labelled & dated rack. Plates should be closed, bagged, labelled & dated. All food to be kept for more than two days should be refrigerated. Don't expect a fan or a screen to keep out loose flies: check.
