# Supplementary figures and images for: Predicting death by the loss of intestinal function
Source: PLoS One. 2020 Apr 14;15(4):e0230970. doi: 10.1371/journal.pone.0230970 (PMC7156097; doi:10.1371/journal.pone.0230970)

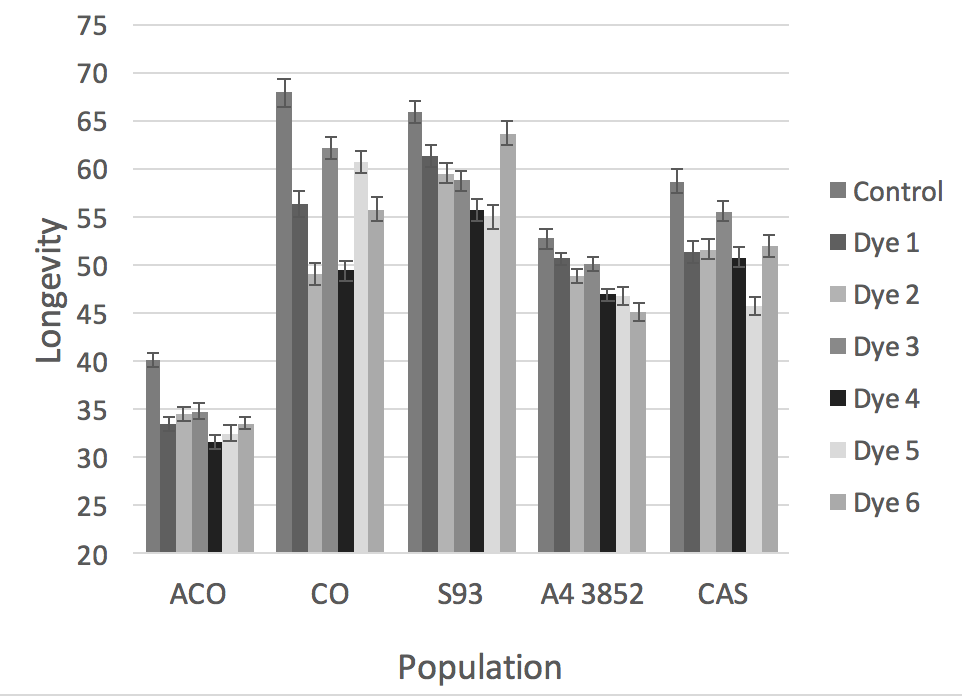

Supplement: S1 Fig — Standard error bars. When pooling the dyes against the control, the control flies lived significantly longer than the flies in an environment with dye in the food. The dyes used: Dye 1: SPS Alfachem Blue, Dye 2: Sigma Aldrich, Dye 3: Spectrum Blue, Dye 4: Flavors and Color Blue, Dye 5: Chemistry Connection Blue, and Dye 6: Electric Blue. (TIF) [file pone.0230970.s002.tif]

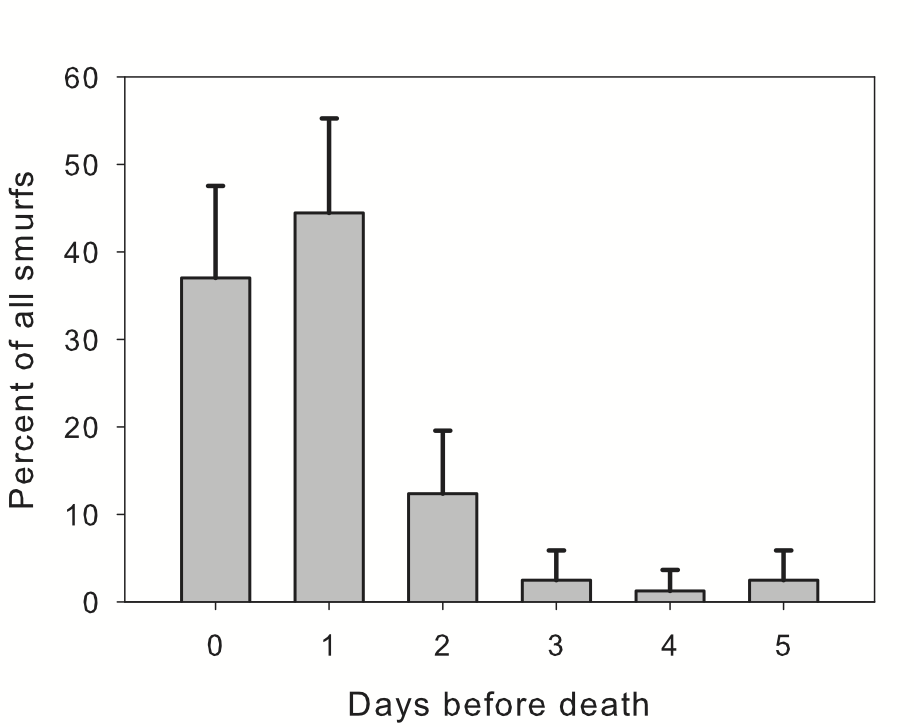

Supplement: S2 Fig — The percent of first appearance of all 81 Smurfs as a function of the days before death when raised on food with dye 1. The bars are simultaneous 95% confidence intervals. The majority became Smurfs on the day they were found dead (day 0) or 1 day before death. A total of 47% (95% confidence interval, (39%, 54%)) eventually became Smurfs. The mean longevity (from egg) of all flies in this experiment was 32.9 days (95% confidence interval ±1.4 days). These results are consistent with those in the full experiment. Specifically, less than 50% of all flies became Smurfs prior to death and those that did become Smurfs most frequently did so on the day or day before they died. (TIF) [file pone.0230970.s003.tif]
