# Supplementary material for: Predicting death by the loss of intestinal function
Source: PLoS One. 2020 Apr 14;15(4):e0230970. doi: 10.1371/journal.pone.0230970 (PMC7156097; doi:10.1371/journal.pone.0230970)
Supplement: S1 Table — Also provided the average day of Smurfing. (DOCX) [file pone.0230970.s004.docx]

Table S1. Average longevity for every population and every dye used in the experiment. Also provided the average day of Smurfing.

| **Population** |  | **Dye 1** | **Dye 2** | **Dye 3** | **Dye 4** | **Dye 5** | **Dye 6** | **Control** |
| --- | --- | --- | --- | --- | --- | --- | --- | --- |
| ACO | Average Day of Death | 33.518 | 34.558 | 34.815 | 31.655 | 32.545 | 33.566 | 40.053 |
|  | Average day of Smurf | 30.129 | 31.143 | 26.571 | 26.8 | 28.913 | 31.2 | N/A |
| CO | Average Day of Death | 56.255 | 49.107 | 62.143 | 49.439 | 60.689 | 55.719 | 67.87 |
|  | Average day of Smurf | 43.538 | 41.944 | 61.857 | 48.545 | 63.143 | 53.929 | N/A |
| S93 | Average Day of Death | 61.293 | 59.508 | 58.724 | 55.712 | 54.95 | 63.678 | 65.86 |
|  | Average day of Smurf | 59.154 | 56.857 | 61.571 | 46.083 | 48.524 | 46.889 | N/A |
| A4 3852 | Average Day of Death | 50.632 | 48.821 | 50.07 | 46.948 | 46.821 | 45.091 | 52.719 |
|  | Average day of Smurf | 47.444 | 49.5 | 50 | 46.5 | 44.739 | 40 | N/A |
| CAS | Average Day of Death | 51.362 | 51.655 | 53.582 | 50.786 | 45.754 | 52.051 | 58.691 |
|  | Average day of Smurf | 50.333 | 54.933 | 45.308 | 46.5 | 41.444 | 41.067 | N/A |
