# Supplementary material for: Predicting death by the loss of intestinal function
Source: PLoS One. 2020 Apr 14;15(4):e0230970. doi: 10.1371/journal.pone.0230970 (PMC7156097; doi:10.1371/journal.pone.0230970)
Supplement: S2 Table — These results are from the R survdiff function in the survival package. The significant results are shown in bold (using the Bonferroni correction for 30 tests). (DOCX) [file pone.0230970.s005.docx]

Table S2. Log-rank *p*-values comparing the control treatment to each of the other populations at each of the dye treatments. These results are from the R *survdiff* function in the *survival* package. The significant results are shown in bold (using the Bonferroni correction for 30 tests).

Population Dye 1 Dye 2 Dye 3 Dye 4 Dye 5 Dye 6

ACO 9.1×10^-3^ 9.4×10^-3^ 4.9×10^-2^ **2.5×10^-4^** 2.0×10^-3^ **3.7×10^-4^**

CO 8.8×10^-3^ **5.9×10^-8^** 4.6×10^-2^ **4.9×10^-8^** 1.1×10^-2^ **3.89×10^-4^**

S93 0.17 3.2×10^-2^ 2.7×10^-2^ 1.6×10^-2^ 1.5×10^-2^ 0.58

A4 3852 1.2×10^-2^ 9.6×10^-3^ 0.051 **2.9×10^-4^** 4.1×10^-3^ 3.4×10^-3^

CAS 3.02×10^-2^ **9.9×10^-4^** 1.01×10^-2^ **4.5×10^-4^** **3.4×10^-6^** 3.2×10^-2^
