# Supplementary material for: Predicting death by the loss of intestinal function
Source: PLoS One. 2020 Apr 14;15(4):e0230970. doi: 10.1371/journal.pone.0230970 (PMC7156097; doi:10.1371/journal.pone.0230970)
Supplement: S3 Table — The median longevity for each population was greater in the control treatment in than every other dye except for A4 3852 and dye 1. (DOCX) [file pone.0230970.s006.docx]

Table S3. The median longevity for each population at every dye treatment. The median longevity for each population was greater in the control treatment in than every other dye except for A4 3852 and dye 1.

Population Dye 1 Dye 2 Dye 3 Dye 4 Dye 5 Dye 6 Control

A4 3852 54 49 51 49 50 47 53

ACO 33 33.5 36 31 33 33 37

CAS 51 54 57 54.5 45 51 63

CO 56 54 65 49 59 55 68

S93 63.5 63 62.5 56 54 68 68
