# Supplementary material for: Predicting death by the loss of intestinal function
Source: PLoS One. 2020 Apr 14;15(4):e0230970. doi: 10.1371/journal.pone.0230970 (PMC7156097; doi:10.1371/journal.pone.0230970)
Supplement: S4 Table — (DOCX) [file pone.0230970.s007.docx]

Table S4. The maximum longevity for each population, dye combination.

Population Dye 1 Dye 2 Dye 3 Dye 4 Dye 5 Dye 6 Control

ACO 62 61 64 63 71 55 74

CO 104 86 105 88 123 95 100

S93 100 100 89 85 100 96 105

A4 3852 70 71 72 65 67 73 86

CAS 89 84 89 82 79 86 89
