# Supplementary material for: Predicting death by the loss of intestinal function
Source: PLoS One. 2020 Apr 14;15(4):e0230970. doi: 10.1371/journal.pone.0230970 (PMC7156097; doi:10.1371/journal.pone.0230970)
Supplement: S5 Table — (DOCX) [file pone.0230970.s008.docx]

Table S5. The number of flies that Smurfed per population and per dye, as well as the number of total flies per population and dye.

| **Population** |  | **Dye 1** | **Dye 2** | **Dye 3** | **Dye 4** | **Dye 5** | **Dye 6** |
| --- | --- | --- | --- | --- | --- | --- | --- |
| **ACO** | **TOTAL Smurf** | 30 | 21 | 14 | 20 | 23 | 15 |
|  | **Total Flies** | 56 | 52 | 54 | 54 | 55 | 53 |
| **CO** | **TOTAL Smurf** | 13 | 18 | 14 | 22 | 14 | 28 |
|  | **Total Flies** | 55 | 56 | 56 | 57 | 61 | 57 |
| **S93** | **TOTAL Smurf** | 13 | 14 | 7 | 12 | 21 | 9 |
|  | **Total Flies** | 58 | 59 | 58 | 59 | 60 | 59 |
| **A4 3852** | **TOTAL Smurf** | 9 | 12 | 11 | 10 | 23 | 17 |
|  | **Total Flies** | 56 | 56 | 57 | 58 | 56 | 55 |
| **CAS** | **TOTAL Smurf** | 12 | 15 | 13 | 14 | 18 | 15 |
|  | **Total Flies** | 58 | 58 | 55 | 56 | 57 | 59 |
