# Supplementary material for: Predicting death by the loss of intestinal function
Source: PLoS One. 2020 Apr 14;15(4):e0230970. doi: 10.1371/journal.pone.0230970 (PMC7156097; doi:10.1371/journal.pone.0230970)
Supplement: Attachment — Submitted filename: Response to Reviewers.docx [file pone.0230970.s009.docx]

Thank you for your time to review our paper. Please see below for the original points mentioned in the review, as well as our responses to each.

1. Although short, the manuscript reports on issues that impact a good number of labs in the field. However as it stands it is difficult to send for review because of formatting issues.

*We have addressed all of the issues and concerns below regarding formatting issues and we are submitting a Revised Manuscript.*

2. Please remove the figure legend text from within teh Results section and add it to the end after the references.

*The figure legend text from within the Results section was moved and placed at the end of the references in a section called Supporting Material.*

3. Same for the tables and their legends. Please move them to the respective figure and legend sections.

*All of the Tables and legends were moved from their current positions to the respective Supporting Material section at the end of the paper after the References, which now contains the tables, their legends, and figure legends.*

4. It is useful if a photo of the "food straw" used was provided.

*A straw photo was inputted into the Revised Manuscript as Figure 1. All other figures were numerically renamed – Figure 1 became Figure 2, Figure 2 became Figure 3, etc.*

Journal Requirements:
1. When submitting your revision, we need you to address these additional requirements.

 Please ensure that your manuscript meets PLOS ONE's style requirements, including those for file naming. The PLOS ONE style templates can be found at

<http://www.journals.plos.org/plosone/s/file?id=wjVg/PLOSOne_formatting_sample_main_body.pdf> and <http://www.journals.plos.org/plosone/s/file?id=ba62/PLOSOne_formatting_sample_title_authors_affiliations.pdf>

*We made sure our paper meets your requirements. All citations were fixed, as well as some minor formatting issues throughout the manuscript. The only part of the paper that is set up differently than your requirements is that our tables, table legends, and figure legends were moved to the end of the paper as was addressed and requested in the original concerns (please see above, points 2 and 3).*

2. We note that you have stated that you will provide repository information for your data at acceptance. Should your manuscript be accepted for publication, we will hold it until you provide the relevant accession numbers or DOIs necessary to access your data. If you wish to make changes to your Data Availability statement, please describe these changes in your cover letter and we will update your Data Availability statement to reflect the information you provide.

*We will upload the data and have it ready at acceptance.*

3. Thank you for stating the following financial disclosure:

"No -The funders had no role in study design, data collection and analysis, decision to publish, or preparation of the manuscript.".

a. Please provide an amended Funding Statement that declares *all* the funding or sources of support received during this specific study (whether external or internal to your organization) as detailed online in our guide for authors at <http://journals.plos.org/plosone/s/submit-now>.

b. Please state what role the funders took in the study.  If any authors received a salary from any of your funders, please state which authors and which funder. If the funders had no role, please state: "The funders had no role in study design, data collection and analysis, decision to publish, or preparation of the manuscript."

* Please include your amended statements within your cover letter; we will change the online submission form on your behalf.
*Regarding the financial disclosure*, *we will include the amended statements in the cover letter.*

[Note: HTML markup is below. Please do not edit.]
Reviewers' comments:

*There were no reviewers comments in this section, therefore we didn’t have anything to address and change other than the original comments above.*
