# Supplementary material for: Predicting death by the loss of intestinal function
Source: PLoS One. 2020 Apr 14;15(4):e0230970. doi: 10.1371/journal.pone.0230970 (PMC7156097; doi:10.1371/journal.pone.0230970)
Supplement: Attachment — Submitted filename: Response to Reviewers.docx [file pone.0230970.s010.docx]

08 January 2020

Efthimios M. C. Skoulakis, PhD

Academic Editor

PLOS ONE

RE: PONE-D-19-29500R1

Dear Dr. Skoulakis:

Thank you for your prompt and helpful reviews of our manuscript “The Efficacy of Predicting Death by the Loss of Intestinal Function,” which has now been renamed as “Predicting Death by Loss of Intestinal Function.” We have revised the manuscript extensively considering the reviewer’s comments. Below we have reproduced those comments and followed them with a short description of our modifications to the paper or our response to the comments.

Thank you for your time and attention.

Sincerely,

Kathreen Bitner

Reviewer’s comments will be in standard font and our reply will be in red italic font.

REVIEWER #1

This is a manuscript reporting on the reliability of using dyes as food supplements to predict death in Drosophila. In the original paper: Rera M, Clarck RI, Walker DW.M. 2012. Intestinal barrier dysfunction links metabolic and inflammatory markers of aging to death in Drosophila. Proc Natl Acad Sci U S A. 2012 Dec 26;109(52):21528-33, has been suggested that intestinal barrier dysfunction predicts impending death in individual flies. The underlying hypothesis here is that when the intestine epithelium brakes down during aging then the dye, consumed with the food, diffuses from the gut to the rest of the body via circulation and thus the fly exhibits full coloration; it becomes smurf.

In contrast, the results of the present manuscript clearly demonstrate that this is not the case. By using different dyes at similar to the Rera et al, 2012 paper concentration the authors demonstrate that only a minor percentage of flies exhibit full body dye coloration (smurfs) and mostly one day prior to death or even the day of death. This suggests that the smurf phenotype is not a useful biomarker of death.

The findings of this manuscript, previously published papers by the group and a recent paper Gaitanidis et al, 2019 (Gaitanidis A., Dimitriadou A., Dowse H., Sanyal S. Duch C., Consoulas C. (2019). Pre-death morbidity in Drosophila and its compression. Aging, 11:1850-1873) demonstrate that apart from the gradual decay during aging, fit flies may collapse and die within a very short time (sudden death). The process has been termed the death spiral or sudden death and can be fast. This is exactly demonstrated in the present manuscript. Few flies become smurf several days prior to death whereas others (the majority) only the day of death.

That said, I firmly believe that this manuscript should be published.

*This is a succinct and accurate summary of the paper.*

203 We

204 do note that the food used in our study has only 2.5 grams of dye per 100 mL of food whereas

205 Rera put 3.12 grams of dye in 100 mL of food [16].

-Please refer to the paper `Rera et al, 2012` than to one author. All authors worked for this publication. Furthermore, in the materials and methods section of the aforementioned paper is clearly mentioned that the dye concentration is 2.5% (wt/vol). Thus, remove or modify your claim.

*We changed the reference throughout the paper to Rera et al 2012 and removed any reference to only “Rera”. We revised the text to reflect that Rera et al. 2012 used food with a 2.5% dye concentration.*

154 the control treatments lived significantly longer then every other treatment.

-Please correct.

*English corrected.*

213 integrity and become Smurfs andthose that don’t, but they fail to provide exact details on how

214 many individuals become s Smurfs prior to death.

*This typo has been corrected.*

-Add and refer to the Gaitanidis et al, 2019 paper (Gaitanidis A., Dimitriadou A., Dowse H., Sanyal S. Duch C., Consoulas C. (2019). Pre-death morbidity in Drosophila and its compression. Aging, 11:1850-1873).

*This reference was added. Thank you very much for the recommendation*

-The manuscript needs proof reading.

*We have done more careful proof reading and consequently made numerous additional corrections to the manuscript.*

REVIEWER #2

This is a very (too) short manuscript that reports an interesting observation but it is lacking a lot of critical information. It is unclear how the experiments were done and it is impossible to replicate the experiments with the information provided.

*We have added expanded discussion of the experimental methods including a detailed recipe for our standard fly food which is included in the appendix.*

*[as well as the graphs that were requested have been created and are provided in the supplemental section of the paper. A new table was created that clarifies the number of flies used per population and per dye, showing that the experiment had replication. The original dye used, SPS Alfachem, was replicated in 5 different populations, and each population was replicated in 6 different dye environments, allowing for substantial replication across the whole experiment.]*

The authors do not indicate what is actually measured, stating only longevity. Is it the average, mean, max longevity?

*We are measuring mean longevity and this has been clarified in the paper.*

It is standard in the field of aging to provide all the data in separate tables (in supplemental data), like for instance Table S1 in PNAS 111(22):8137,2014.

*As indicated on the cover page we will deposit the raw data in DRYAD upon acceptance of the paper.*

It is IMPERATIVE that the authors provide tables containing each experimental replicate for each dye and control with the corresponding sample size, average, median and max longevity, and logrank P values (% smurfs included but a second table would be better to be able to include all daily scorings done).

*We now have tables in the main part of the paper or in a supplement which give details for each population and dye combination, sample sizes and mean longevities. We have not included the median, maximum longevity or logrank p-values. We did no analyses of the median or maximum longevity. Likewise, since we do not have censored data, we can explore the effects of population and dye more effectively with a standard linear model which we have done. Certainly, with our raw data available any interested scientist can do additional analyses of our data*.

Table 1 and 2 are very confusing and strongly suggest that the experiment have been done only once. The material and methods indicates that cohorts of about 56 flies were used but table 1 indicates for example that ~280 flies were exposed per Dye/Control which means that 56 flies from each population was used and based on the number used for each population in table 2 (for instance ACO) 383 flies is the number of flies needed for one experiment (control + 6 dyes: 56*7=392).

*We created a new table (Table 1) that clarifies the number of flies used per population and per dye in the experiment, as well as the number of males and females used. As can be seen from the table, there was substantial replication, with each population undergoing 6 different dyes and a control. The original dye used, SPS Alfachem, was replicated in 5 different populations, and each population was replicated in 6 different dye environments, allowing for substantial replication across the whole experiment. Most importantly the major results we reach are consistent across the five different populations and six different dyes. It is likely that repeating these experiments in a different month or year would not yield the exact results. However, the variation in this experiment due to fly populations that vary tremendously in levels of inbreeding and evolutionary history and the use of many different provides a robust testing regime.*

It is also suspicious that figure 2 has error bars (no explanation in legend, is it SD, SE?) that are all exactly identical. This article cannot be published and I cannot properly evaluate it until the authors provide complete experimental details and data, and that the experiment has been replicated.

*The figure legend now clarifies that the bars are 95% confidence intervals that were estimated by the R function emmeans. This function uses a pooled estimate of variance. Since the degrees of freedom are similar for each dye the confidence intervals look similar in size. This pooling is not unreasonable given the similarity in longevities.*

The gender of the animals tested is also not provided but the statistical analysis section of the material and method does indicate that males and females were used.

*This has been clarified in multiple places in the paper most prominently in Table 1. There were roughly equal numbers of males and females used in the experiment.*

The authors performed an important control experiment to see if tapping affect longevity yet do not provide any data/figure from these experiments nor it is mentioned in the result, yet the discussion states on line 177 that the experiment show no detectable effects of taping. There is no justification to why the authors perform this test only with ACO females while the subsequent experiments use both gender and 4 additional genotypes.

*We have clarified that both males and females were used in this experiment. The exact numbers of flies used for the Tapping experiment are now described in the Materials and Methods. The results of the tapping experiment are summarized in a new Table 2 and discussed in the Results section.*

The authors do not report how the flies were collected (anesthesia or not, kind of anesthesia, duration of anesthesia and time window/age interval).

*This has been clarified in detail in the methods. The flies were knocked out using CO_2_, separated by gender and placed into the individual straw containers.*

The authors do not indicate how and if they ensure that each fly has actually consumed the food. From the material and method, I guess that the authors did check coloring of the gut but should clearly indicates that all flies did show a blue gut. It remains possible that the proportion of flies that are not “smurfing” consumed less food and consequently not enough dye is ingested to display color outside of the gut.

*While it is true we have no way knowing exactly if the flies consumed the food, this technique consisted of putting dye in the food and following body color changes. There might be variation in how much food a fly consumes, but since each fly was in their own environment with only the dyed food, the flies had no other option but to consume the food or die from starvation. This was already clarified in the materials and methods section regarding the fact that all flies did have a blue gut: “Smurf status required that the entire body changed to any variation of a blue color. This was an important distinction as Drosophila flies fed food with a blue dye would have visible blue coloring in only the gut portion when they weren’t a Smurf.” In this paper we do not try to assess why the technique fails, uneven food consumption is one possibility. But at the present time the method relies on the flies consuming the food with dye.*

The composition of the fly food used is not provided.

*The food used is a banana molasses recipe that has been used in our lab for over 3 decades. The detailed recipe is given in the supplementary material*

Figure 1 and 4 should be combined together (method of delivery/monitoring, example of smurf flies).

*The two figures are now combined together as requested.*
