# Supplementary material for: Predicting death by the loss of intestinal function
Source: PLoS One. 2020 Apr 14;15(4):e0230970. doi: 10.1371/journal.pone.0230970 (PMC7156097; doi:10.1371/journal.pone.0230970)
Supplement: Attachment — Submitted filename: Response to Reviewers.docx [file pone.0230970.s011.docx]

3 March 2020

Efthimios M. C. Skoulakis, PhD

Academic Editor

PLOS ONE

RE: PONE-D-19-29500R2

Predicting Death by the Loss of Intestinal Function

Dear Dr. Skoulakis,

Thank you for your latest (23 January 2020) comments and referee reports on our above referenced paper. We have undertaken a number of revisions which we detail below. In addition we have provided the raw data from our experiment for the referees to review. When the paper is accepted these data will be posted on the public site Dryad. Finally we have added to the supplement section data from a preliminary experiment that is consistent with the major observations show in our primary experiment – that a minority of flies become Smurfs before death and most do so the day of their death or one day before death.

We appreciate your receptivity to our additional revisions.

Sincerely,

Laurence D. Mueller

Kathreen Bitner

Below we reproduce the comments of each referee in normal font and our responses are shown in italic font.

Reviewer #1

All questions were addressed by the authors. This is a useful manuscript uncovering limitations of a technique used to predict death in flies. Conceptually, intestinal integrity decay during aging may not be the most common pathway to death in flies.

*We appreciate the referee’s comments and would not anticipate that any of our new edits and material would change his/her mind.*

Reviewer #2

The authors have largely addressed the comments of the reviewers and provided the technical details required for one to replicate the experiments, however, it remains very concerning that the authors still do not provide critical information to properly review this manuscript (max longevity and log rank p values). It is especially troubling that the authors still do not provide access to the data and respond with “we will deposit the raw data in DRYAD upon acceptance of the paper”. The review of scientific data must be done before an article is published and should not be provided upon acceptance of the paper.

*We have now provided in the supplementary section of the manuscript, the maximum longevity by populations and dye treatment, the median longevity by population and dye treatment and the log rank p values by population and dye treatment. The log rank p values actually reveal more significant differences than do the comparisons of mean longevity. In addition, with these revisions we have submitted a separate file with the raw data from the main experiment and from a preliminary experiment. When the paper is accepted for publication, we still intend to place the raw data on the Dryad website.*

It is now clear that none of the experiments have been experimentally replicated and although the statistical analysis allows for “substantial replicate” it is not sufficient to ensure that the results presented are reproducible.

*In this revision we have included the results from a preliminary study of the ACO population and dye #1. This preliminary study supports our most important conclusions, (i) the majority of flies never become Smurfs, (ii) those that do become Smurfs typically do so just before death.*

The authors responded that “We did no analyses of the median or maximum longevity” yet states in the article: ”there were significant differences in mean longevity between all populations and their controls except CO and s93 (p=0.16”. For a given population, only one measurement (only one experimental replicate) is made with less than 30 individuals (per gender).

*While it is the case that our experiment consisted of only one experiment with a particular population and dye this observation ignores the results of each population that was tested multiple times with six different dyes. This is important since each dye has the same general effect, (i) a minority of flies becoming Smurfs, (ii) those that do become Smurfs do so shortly before death, (iii) longevity is reduced. So, in our assessment, and we think for many others, the broad consistency and* ***replication*** *of results (i-iii) over different specific dyes and different populations is the important and robust message. These conclusions draw their strength precisely because they have been replicated in so many different situations.*

The statistical validation of the experiment does not mean that the experiment is reproducible and certainly does not warrant that the resulting interpretation is biologically correct. The need for independent experimental replicate of those experiments is especially needed when the ACO longevity is obviously different between the tapping experiment (53-55 days) and the dye experiment (40 days).

*By these comments it is apparent the referee is most concerned with replication at different calendar times. We have already alluded to the fact that our main conclusions are supported by replication of different populations and dyes. We also have alluded that the preliminary study, while not replicating the entire experiment, was done at a different calendar date and did give results consistent with the main experiment. The referee points to the different absolute longevities for ACO flies in the tapping experiment and the main experiment. Firstly, for assessing whether tapping effects longevity, the absolute longevity differences do not interfere with our ability to draw conclusions. Secondly, we suspect the referee is making a more subtle suggestion that measurement can change in unpredictable ways in our lab when done at different times. Although I don’t think it is important to understand why longevity might change from one experiment to another, these block effects are observed often in populations we do repeated longevity assays on, and there are several explanations. One that is specific to this experiment is the fact that flies were kept individually in straws until death. However, prior to being placed in straws they were in cultures with many flies of both sexes and some were old enough to mate. However, once placed in straws they could no longer mate and would thus eventually experience a reduction in mortality from the absence of mating (Partridge et al., 1986, J. Insect Physio. 32:925). The relative impact of this reduction in mating would certainly be a function of how many of the flies had mated prior to their isolation in the straws and this could have certainly varied from one experiment to another.*

The lack of experimental replicate also applies to the tapping experiment that the authors have now clearly stated that they did it “to see if our methods for checking for Smurf flies would affect the mean longevity of the fly”. In this case the authors also uses an inappropriate method (t test). The authors still do not justify why they only tested the effect of taping only on the ACO population when the data show obvious differences in longevity of controls (and differences between control and dye treatments are not always significant). It cannot be concluded that the absence of effect on longevity by tapping of the ACO individuals would show that the taping has no effect on the longevity of the other populations.

*The referee states that a t-test on the means of two large samples of independent observations is inappropriate although the referee provides no justification for that conclusion. I will add our justification. It is the central limit theorem which can be used to show the asymptotic normal distribution of the sample mean, which is independent of the underlying random variables distribution. Since our concern was with the mean longevity decline observed when flies were cultured with dyes testing the effect of tapping on the mean longevity is precisely what should have been done. We understand that by not conducting a tapping experiment under all conditions there is the possibility of alternative outcomes. Thus, we have added the following statement to our “Conclusions” section: “We only tested one population, ACO, for an effect of tapping on mean longevity. Thus, for the ACO population it is clear that dyes are responsible for their reduced longevity not tapping. While it is theoretically possible that the other populations are not affected by the dyes but are affected by tapping, we believe this is an unlikely possibility.”*

In the absence of experimental replicates, I am not confident that the data presented are reproducible and that the biological conclusions are scientifically valid and therefore I cannot recommend to accept this manuscript for publication.

*As stated, before we believe that there is in fact replication that provides a consistent picture of the effect of dyes on producing the Smurf phenotype. We believe this is an important contribution since there has been no published study with this level of detail about this potentially important technique.*
